# Ischemic lumbosacral plexopathy after embolization of type 2 endoleak: Progress and functional outcome

**DOI:** 10.1002/ccr3.5182

**Published:** 2021-12-07

**Authors:** Kuan Geok Ng, Derek Chunyin Ho, Tze Chao Wee

**Affiliations:** ^1^ Department of General Medicine/Rehabilitation Medicine Sengkang General Hospital Singapore City Singapore; ^2^ Department of General Surgery Changi General Hospital Singapore City Singapore; ^3^ Department of Rehabilitation Medicine Changi General Hospital Singapore City Singapore

**Keywords:** embolization, ischemic lumbosacral plexopathy, outcome, rehabilitation

## Abstract

An endoleak is a complication that can occur after an endovascular aneurysm repair. We report a rare case of ischemic lumbosacral plexopathy post embolization of type 2 endoleak, including its presentation, neurological progress, rehabilitation strategy and functional outcome.

## Graphical abstract

Ischemic lumbosacral plexopathy is a devastating complication, and rehabilitation is essential to optimize function. More research is required to further refine the evidence for rehabilitation of lesions involving peripheral nervous system.

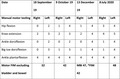

## 1 INTRODUCTION

Peripheral nerve injuries, including plexopathy, are an important cause of disability with the neurological sequelae affecting activities of daily living and quality of life. Lumbosacral plexopathy represents a distinct entity of peripheral nerve disorders due to its relative rarity.

The lumbosacral plexus represents the nerve supply to the lower back, pelvis, and legs and is derived from the ventral rami of the L1‐S4 nerve roots.^1^ Common etiologies for lumbosacral plexopathy including diabetes, neoplastic invasion, radiation, trauma, infection, inflammatory, infiltrative, and vascular causes like ischemia, hemorrhage, and direct compression by aortoiliac aneurysm.^1^

The lumbosacral plexus has a very rich vascular supply from the five lumbar arteries that originate from the abdominal aorta, the deep circumflex iliac artery that branches from the external iliac artery, and the iliolumbar and gluteal branches of the internal iliac artery.^2^ Due to its rich vascular supply, ischemia of the plexus is rather unusual. However, ischemic lumbosacral plexopathy has been reported with aortoiliac procedure.^3^ The overall neurological risk for endovascular and open abdominal aortic surgery ranges between 0 and 1%.^4^ Neurological complication of nonselective or inadvertent embolization of lumbar arteries is extremely rare but is a known complication.^5^

We report a case of ischemic lumbosacral plexopathy in a woman who underwent selective embolization of type 2 endoleak.

## 2 CASE REPORT

A patient in her late seventies was incidentally found to have a large infrarenal abdominal aortic aneurysm in 2013 for which she had undergone an uneventful percutaneous endovascular aortic repair with left chimney. She lived alone, remained independent in her activities of daily living, and was community ambulant without aid.

She was electively admitted in September 2019 for embolization of a type 2 endoleak by the interventional radiologist. Super selective cannulation of the distal aspect of the iliolumbar branch supplying the nidus with a micro catheter was performed. Embolization was carried out using Onyx^®^ until complete exclusion of the nidus. This was followed by repeat aortogram, which showed complete exclusion of the nidus from right‐sided branches. However, there was continued filling of the nidus from the left lumbar branches. It was therefore decided to embolize the left side. After embolization, angiogram showed complete exclusion of the endoleak and the left‐sided branches supplying the endo leak.

Five hours after the procedure the patient complained of bilateral lower limb weakness and numbness, right more than left. Physical examination revealed lower motor neurone pattern of weakness over bilateral lower limbs, right worse than left (Table 1). Sensation testing revealed normal sensation over left, impaired sensation for right L2 to S1. Proprioception at bilateral big toes was intact. Reflexes were absent in bilateral lower limbs.

**TABLE 1:** Progress of patient's manual motor testing power and motor functional independence measure (FIM)

Date	September 18, 2019		October 9, 2019		December 13, 2019		July 8, 2020	
Manual motor testing	Right	Left	Right	Left	Right	Left	Right	Left
Hip flexion	0	2	1	4	1	4	3	4
Knee extension	2	3	2	3	2	4	4	5
Ankle dorsiflexion	0	2	0	2	0	4	1	4
Big toe dorsiflexion	0	3	1	3	1	4	1	4
Ankle plantarflexion	4	4	4	4	4	4	4	4
Motor FIM excluding bladder and bowel	32		42		MBI 47, ^a^FIM 42			48

^a^ For inpatient rehabilitation, we used FIM to assess patient's function and track patient's recovery trajectory. However in community hospital, modified Barthel index (MBI) commonly used. We have walked‐over patient's MBI to FIM based on study conducted by Ickpyo Hong et al.^21^

Lumbar spinal drain was inserted to decompress the spinal cord to allow more arterial flow as there was concern with spinal cord ischemia. The patient was started on fluid replacement to maintain the mean arterial pressure above 80 mm Hg. Urgent CT aortogram followed by MRI thoracolumbar spine was performed. Aortogram showed postinterval embolization of bilateral feeding arteries. Onyx material was seen within a branch of the embolized right iliolumbar artery, which extends into the spinal canal at the level of L3 and appears to exit at the level of L2. It ran external to the thecal sac. MRI thoracolumbosacral spine showed no abnormal cord signal or restricted diffusion.

She was subsequently admitted for inpatient multidisciplinary rehabilitation. On initial functional assessment, she required moderate assistance for bed mobility and transfer with poor dynamic sitting and standing balance. She did not complain of pain. After 1 month of inpatient rehabilitation, she made some neurological recovery with functional improvement. She had good dynamic sitting balance and was able to transfer under supervision. She can self‐propel wheelchair within the ward. However, she had poor standing balance and was still unable to ambulate. She was discharged to a subacute rehabilitation facility before being discharged home. Please see her functional improvement over time using the Functional Independence Measure (FIM) instrument illustrated in Table 1.

Electrophysiology study was performed 12 weeks later showed electrophysiological evidence of an acute right lumbar plexopathy involving the right obturator and femoral nerves, with no evidence of axonal continuity.

She was reviewed in the outpatient clinic 14 weeks later. Neurologically remained largely unchanged for right lower limb; however, left lower limb power had further improved to about 4 on manual motor testing. Functionally, she had also made slight progression, and she was able to walk 25 meters using walking frame with 1 person providing standby assist. However, she was still dependent on wheelchair for longer distance ambulation.

## 3 DISCUSSION

We report a rare case of lumbosacral plexopathy after embolization of type 2 endoleak. Endoleak is defined as persistent flow of blood into the aneurysm sac after device placement and indicates a failure to completely exclude the aneurysm. There are 5 types of endoleaks.^6^ Type 2 endoleak results from flow into and out of the aneurysm sac from one or more patent branch vessels.^6^ There is evidence to suggest that endoleak may be associated with an increased risk of rupture.^7^ The decision to intervene for our patient was due to the increasing sac size, which was 7cm.

Onyx^®^ ^8^ is “a non‐adhesive liquid embolic agent comprised of ethylene vinyl alcohol copolymer dissolved in dimethyl sulfoxide, and suspended micronized tantalum powder to provide contrast for visualization under fluoroscopy.” It is not the routine choice for embolization. The most common choice is embolization coil. The advantage of Onyx^®^ is that it can follow the course of small branches of vessels without cannulation. The disadvantage is that it is hard to control the flow and volume needed, and it may flow to unwanted areas. Our patient had aortic repair 7 years ago, and the forward flow via the lumbar arteries from the aorta was already excluded. Her lumbar plexus blood supply was likely dependent on collateral flow, and these collaterals were largely blocked off likely due to back flow of the embolic agent.

Ischemic lumbosacral plexopathy after selective Onyx^®^ embolization is a rare occurrence, and to our knowledge, one case report with similar etiology had been reported in the literature.^5^ In that case, Onyx^®^ liquid embolic agent was injected from the lumbar arteries into the sac, and extensive nontargeted embolization within the iliolumbar arterial plexus occurred. The patient was subsequently noted to have significant weakness of his left lower limb along with sensory deficits. The reported case was managed similarly with bed rest and lumbar drainage.

Diagnosis of lumbosacral plexopathy can be challenging. Clinical history is essential for diagnosis as physical examination cannot delineate the cause of lumbosacral plexopathy. It often requires a combination of clinical, radiological, and electrophysiological test. Main differential diagnosis being lumbosacral polyradiculopathy as clinically both has lower motor neurone pattern of weakness. Frequently, the only way to accurately differentiate them is through electrophysiological study. For lumbosacral polyradiculopathy, as it is a preganglionic lesion, nerve conduction study (NCS) is often normal. Electromyography (EMG) of paraspinal muscle will be abnormal depending on the nerve root involved. Conversely, lumbosacral plexopathy usually presents with abnormal NCS and EMG depending on the nerves involved with a normal paraspinal EMG.

Prognosis of lumbosacral plexopathy is largely depending on the etiology. Traumatic and radiation etiology usually have a poor prognosis, whereas inflammatory and diabetic etiology have a good prognosis if treat promptly.^9^, ^10^ Traumatic lumbosacral plexopathy is usually associated with severe trauma resulting in nerve disruption and radiation usually causes irreversible damage to the nerve, hence resulting in a poorer prognosis.^9^ Pathogenesis of diabetes lumbosacral plexopathy is not fully understood, yet studies have suggested possible immune‐mediated inflammatory microvasculitis.^10^ Inflammatory causes and diabetes both have better prognosis as these cases are usually monophasic illnesses, with prompt diagnosis and treatment with immunotherapy‐like intravenous methylprednisolone, it may stop the inflammatory processes and hasten recovery.^9^, ^10^ Ischemic etiology seems to have a poor prognosis with permanent neurological disability. In a case series, 3 out of 4 cases of ischemic neuropathy of lumbosacral plexus following aortoiliac procedure have significant weakness and disability.^3^ One patient required a frame for ambulation, and the other two patients were wheelchair bound.

There is limited evidence available to guide rehabilitation of patients with lumbosacral plexopathy. Core rehabilitation principles commonly used in this group of patient, which including strengthening, address muscular imbalances, maintain flexibility, and improve balance and gait. Assistive devices and braces as indicated, for example, ankle‐foot orthoses (AFOs) for foot drop. We can potentially extrapolate some rehabilitation strategies from peripheral neuropathy considering plexuses are part of peripheral nervous system. A 2014 systematic review found balance exercises to have the highest effect on motor as well as sensory symptoms in all types of peripheral neuropathy.^11^ Studies focusing exclusively on strength, or a combination of endurance and strength, appeared to have a lower impact. Balance exercises improved both parameters of balance control and gait. Strength and endurance training, not including any balance indices, only achieved improvements regarding muscle atrophy in general like improvement on knee torques and walking capacity. The underlying mechanisms for the beneficial effects of exercise on peripheral neuropathy are not fully understood. Explanations include positive modulation of regenerative mechanisms such as altered expression of growth factors, induction of remyelination, and accelerating axonal regeneration.^12^, ^13^ Treadmill exercise had demonstrated the potential to improve the regeneration of transected nerves by altered expression of neurotrophic growth factors such as nerve growth factor.^14^ There is little evidence for the beneficial effect of nutritional supplements such as vitamin E or high‐dose vitamin B.^15^

Promising therapeutic effect of peripheral magnetic stimulation on traumatic brachial plexopathy had been reported.^16^ Patients were randomized to receive conventional physical therapy modalities and active exercises as well as real or sham repetitive magnetic stimulation (rMS) over superior trapezius muscle of the affected limb daily for 10 sessions. Significant improvement was observed for both muscle strength and shoulder pain in both clinical and neurophysiological parameters. The mechanisms of the response to rMS are still uncertain and potentially work at both spinal and supraspinal levels.^16^ Apart from rMS, there are also few case reports on use of robotic devices for brachial plexus injury post‐surgical repair, which have shown to be effective and safe.^17^, ^18^ However to date, there is no evidence for use of robotic device in lumbosacral plexopathy.

Other than functional deficit, neuropathic pain is another common morbidity in patients affected by plexopathy.^9^ Neuropathic pain management includes pharmacological and nonpharmacological strategies.^19^ For pharmacology treatment, first‐line treatment including tricyclic antidepressants, serotonin‐noradrenaline reuptake inhibitors, pregabalin, and gabapentin.^20^

Our patient had undergone traditional rehabilitation with both restorative and compensatory approaches. She had strengthening exercises for partially affected muscles, range of motion exercises to maintain her flexibility, balance, and gait training with aids. We could potentially further enhance her recovery by trial of robotic gait training and rTMS as both had shown promising results in rehabilitation of peripheral nerve injury.

The main limitation for our case report is limited follow‐up. We only managed to review her twice at 3 months and 9 months postdischarge. We do not have information on her long‐term outcome beyond 9 months. Second, more precise outcome measures such as gait analysis may be used to quantify functional recovery.

## 4 CONCLUSION

Ischemic lumbar plexopathy following embolization of type 2 endoleak is rare and can result in permanent neurological disability. It is a significant complication in which patients need to be aware of. Rehabilitation helps with functional recovery but limited role in neurological recovery.

## ACKNOWLEDGEMENT

The authors recieved no financial support for the authorship, and/or publication of this article.

## CONFLICT OF INTEREST

All authors have no conflicts of interest to declare. This material is the authors' own original work, which has not been previously published elsewhere.

## AUTHOR CONTRIBUTIONS

All authors have been personally and actively involved in substantial work leading to the paper. Dr. Ng KG and Dr. Wee TC recruited patient. Dr. NG KG prepared the manuscript with important intellectual input from Dr. Wee TC and Dr. Derek Ho. All authors approved the final manuscript.

## CONSENT

Written informed consent was obtained from patient to publish this report in accordance with the journal's patient consent policy.

## Data Availability

Data sharing is not applicable to this article as no datasets were generated or analyzed.
